# Reduction of *Escherichia coli* O157:H7 in Finishing Cattle Fed Corn Genetically Modified to Produce Increased Concentrations of Alpha Amylase in the Corn Kernel

**DOI:** 10.1089/fpd.2023.0007

**Published:** 2023-10-03

**Authors:** Joshua M. Maher, James S. Drouillard, Adrian N. Baker, Vanessa de Aguiar Veloso, Qing Kang, Justin J. Kastner, Sara E. Gragg

**Affiliations:** ^1^ Department of Animal Sciences and Industry, Kansas State University, Manhattan, Kansas, USA.; ^2^ Department of Statistics, and Kansas State University, Manhattan, Kansas, USA.; ^3^ Department of Diagnostic Medicine and Pathobiology, Kansas State University, Manhattan, Kansas, USA.

**Keywords:** *Escherichia coli* O157:H7, food safety, feedlot cattle, interventions, amylase, GMO

## Abstract

Cattle are recognized as the principal reservoir for *Escherichia coli* O157:H7 and preharvest food safety efforts often focus on decreasing shedding of this pathogen in cattle feces. Enogen^®^ corn (EC; Syngenta Seeds, LLC) is genetically modified to produce enhanced concentrations of α-amylase in the corn kernel endosperm. Research has demonstrated improvements in feed efficiency for cattle fed EC and research has not yet explored whether improved digestion impacts foodborne pathogen populations in cattle. Therefore, this study explored effects of finishing diets containing EC on *Escherichia coli* O157:H7 prevalence in cattle. A 2 × 2 factorial experiment was conducted with steers (*n* = 960) fed diets consisting of 2 types of silage (EC or Control) and grain (EC or Control), fed daily *ad libitum*. Steers were grouped into 12 blocks by incoming body weight, blocks were randomly assigned to one of four pens, and pens were randomly assigned to one diet. Cattle were sampled using rectoanal mucosal swabs in cohorts of 298–337 cattle per day, for a total of 3 sampling days (15–16 days apart). *Escherichia coli* O157:H7 prevalence rates ranged from not detected (0/75) to 10.0% (8/80) depending on sampling day. Tests for the silage × corn interaction, and the main effects of silage and corn, were not significant (*p* > 0.05); however, EC reduced the odds of *Escherichia coli* O157:H7 prevalence by 43% compared to the control corn diet (*p* = 0.07). Diets containing EC tended to decrease *Escherichia coli* O157:H7 prevalence in feedlot cattle; however, this reduction was not significant. Before a conclusion can be drawn about impact of EC on *Escherichia coli* O157:H7 in cattle, further research is necessary to (1) determine if this tendency is due to increased alpha amylase activity and (2) elucidate impact on *Escherichia coli* O157:H7 prevalence and concentration, as well as a possible mechanism of action.

## Introduction

Estimated to cause over 63,000 foodborne illnesses annually, *Escherichia coli* O157:H7 represents a significant public health threat (Mead et al., 1999; Scallan et al., 2011). Cattle are recognized as the principal reservoir for *Escherichia coli* O157:H7 (Chapman et al., 1993; Hancock et al., 1997; McDaniel et al., 2014; Renwick et al., 1993; Whipp et al., 1994) and asymptomatic carriers, with studies showing up to 30% of cattle harboring the pathogen (Callaway et al., 2009; Callaway et al., 2006; Reinstein et al., 2007; Stanford et al., 2005). The U.S. Food and Drug Administration, Centers for Disease Control and Prevention, and Department of Agriculture surveyed foodborne illness data from 1998 to 2017 and determined that 25.8% of foodborne *Escherichia coli* O157:H7 illnesses were attributable to beef (Interagency Food Safety Analytics Collaboration, 2019).

Within the feedlot segment of U.S. cattle production, animals are routinely fed diets containing high concentrations of grain to optimize feed efficiency (Huntington, 1997; National Academies of Sciences, Engineering, and Medicine, 2016). Ruminants have evolved to eat cellulose-containing plant material and the rumen microbiota can also degrade starch (Huntington, 1997; Huntington et al., 2006; Owens et al., 1986; Westreicher-Kristen et al., 2018). Corn, the most widely utilized source of grain in U.S. cattle finishing diets (Samuelson et al., 2016), primarily comprises starch.

Increased starch digestion maximizes cattle feeding efficiency for feedlot producers. Enogen^®^ corn (EC; Syngenta Seeds, LLC), which was launched in 2011, contains a thermotolerant α-amylase enzyme trait that improves cattle performance by potentially modifying site of starch digestion, as cattle fed dry-rolled EC corn were associated with a numerical increase in postruminal and total tract digestibility of starch (Jolly-Breithaupt et al., 2016). Research conducted by Horton et al. (2020) suggests that steam flaking EC may enhance activity of the thermotolerant α-amylase enzyme, and steam flaking EC improved feed efficiency by ∼5% when EC was flaked to 390 g/L compared to conventional (CON) corn flaked to 360 g/L.

Similarly, Johnson et al. (2019) observed improvements in feed efficiency in growing cattle diets when EC was fed as corn silage. Research evaluating the impact of EC on site and extent of starch digestion is limited. Total tract digestion of steam flaked corn (SFC) is ∼99% and therefore a 5% improvement in feed efficiency suggests EC alters site of starch digestion (Owens and Zinn, 2005). Research on the food safety implications of modifying cattle diets is well documented in the literature; however, the impact of feeding EC on foodborne pathogen shedding has not been evaluated. Addressing this knowledge gap by evaluating the impact of feeding EC as SFC and corn silage on *Escherichia coli* O157:H7 prevalence in finishing cattle is the objective of this study.

## Materials and Methods

All procedures followed in this study were approved by the Kansas State University Institutional Animal Care and Use Committee and the Institutional Biosafety Committee.

### Experimental design

A randomized complete block experiment was conducted with steers (*n* = 960; 388 ± 7.4 kg initial body weight) grouped into 12 blocks by incoming body weight. Steers within each body weight block were assigned at random to one of four pens, which were assigned to one of 4 diets at random.

Cattle were fed diets consisting of two types of silage (EC or Control) and two types of corn (EC or Control) in a 2 × 2 factorial arrangement of treatments. Each diet was applied to 12 pens containing 15 or 25 steers per pen (6 pens containing 15 steers and 6 pens containing 25 steers); therefore, each dietary treatment was supplied to 240 cattle in total. Prevalence of *Escherichia coli* O157:H7 was measured using rectoanal mucosal swabs (RAMS) from each animal. Cattle were sampled in cohorts of 298–337 cattle per day, for a total of 3 sampling days (15–16 days apart, sampling dates identified below in *Cattle Diets*), yielding 955 RAMS samples. A RAMS sample was not collected from a total of five animals originally on trial. Cattle were sampled at the Beef Cattle Research Center (BCRC) of Kansas State University.

All cattle were fed finishing diets during the finishing period, and each animal was sampled one time throughout the feeding period. Animals were sampled in accordance with the block to which they were assigned, such that animals from a single block were all sampled on the same day. Samples were collected after 62 (sampling day 1; June 12, 2018), 78 (sampling day 2; June 28, 2018), and 93 (sampling day 3; July 13, 2018) days. These sampling dates coincided with re-implanting the steers based on harvest days to achieve similar days on final implant before harvest. Steers were not tested for *Escherichia coli* O157:H7 carriage before the study.

### Cattle diets

The finishing diet treatments were as follows: control silage with control corn, Enogen silage with control corn, control silage with Enogen corn, and Enogen silage with Enogen corn. EC (Syngenta Seeds, LLC) was sourced from the study sponsor, while CON corn was sourced locally. Corn was conditioned by adding moisture and surfactant (SarTemp; SarTec, Anoka, MN, USA), steam conditioned for 40 min, and flaked daily utilizing an R&R Machine Works steam-flaker (46 × 91 cm corrugated rolls; Dalhart, TX, USA).

The CON corn was flaked to a bulk density of 360 g/L, while EC was flaked to a bulk density of 390 g/L to achieve similar starch availability of flaked grains, as described by Horton et al. (2020), to determine if the α-amylase activity in EC corn impacts digestibility in comparison to control corn with the same starch availability. Analysis of starch availability was performed daily using the method described by Sindt et al. (2006). Corn silages were planted at Kansas State University and harvested using a commercial harvester (John Deere, Model 7980; Moline, IL, USA). Whole chopped corn plant was transported to the BCRC and packed into agricultural bags (Hitec Bag; Plastika Kritis, Iraklion, Greece) for ensiling and storage.

Composition of finishing diets (Table 1), including silage (Table 2) and grain (Table 3), was analyzed for nutrient composition using monthly composite of samples for each ingredient (SDK Labs, Hutchinson, KS, USA). Animals were fed once daily *ad libitum*. Feed efficiency and animal performance metrics were evaluated during this study, as outlined by the companion study (Baker et al., 2019).

**Table 1.** Finishing Diet Composition Fed to Steers for 62 (Sampling Day 1), 78 (Sampling Day 2), and 93 (Sampling Day 3)^a^ Days During the Finishing Period

Composition of finishing diets fed to steers^b^				
Item	Control silage		Enogen^®^ silage	
	Control grain	Enogen grain	Control grain	Enogen grain
Ingredient, % of DM				
Control steam-flaked corn	74.5	0.0	74.5	0.0
Enogen steam-flaked corn	0.0	74.5	0.0	74.5
Control corn silage	8.0	8.0	0.0	0.0
Enogen corn silage	0.0	0.0	8.0	8.0
Ground alfalfa	2.0	2.0	2.0	2.0
Sweet Bran^®^	12.0	12.0	12.0	12.0
Supplement^c^	3.5	3.5	3.5	3.5
Analyzed composition,^d^ % of diet DM				
Crude protein	14.04	14.26	13.89	14.11
Acid detergent fiber	7.43	8.31	6.79	7.58
Ether extract	2.96	4.34	3.00	4.39
Calcium	0.71	0.72	0.71	0.72
Phosphorous	0.29	0.37	0.28	0.37
Potassium	0.75	0.87	0.68	0.80

^a^ Animals were sampled in cohorts of 300–340 animals on 3 sampling days (June 12, 2018, June 28, 2018, and July 13, 2018).

^b^ Diets were top dressed with 400 mg/animal daily of ractopamine (Optaflexx, Elanco Animal Health, Greenfield, IN, USA) in a ground corn carrier for 35 days before harvest.

^c^ Consisted of urea, salt, limestone, trace mineral premix, and potassium chloride, and provided 36.4 mg/kg monensin (Rumensin; Elanco Animal Health) in the total diet DM.

^d^ Analyzed nutrient composition of ingredients in diet by SDK Labs (Hutchinson, KS, USA). Weekly samples were dried in a 55°C oven and composited on an equal weight basis within each month (6 samples per ingredient).

DM, dry matter.

**Table 2.** Silage Analyzed Nutrients and Composition Fed to Steers for 62 (Sampling Day 1), 78 (Sampling Day 2), and 93 (Sampling Day 3) Days During the Finishing Period

Composition of silages fed to steers		
Item, % of DM	Control silage	Enogen^®^ silage
Dry matter	26.96	35.67
Crude protein	9.15	7.26
Acid detergent fiber	32.78	22.15
Neutral detergent fiber	57.47	38.21
Ether extract	2.04	2.63
Calcium	0.39	0.30
Phosphorous	0.24	0.19
Potassium	2.28	1.29
Starch	2.79	30.73
Ash	7.73	4.88
pH	3.93	3.78

**Table 3.** Nutrient Composition of Steam-Flaked Enogen Corn and Control Corn Fed to Steers for 62 (Sampling Day 1), 78 (Sampling Day 2), and 93 (Sampling Day 3) Days During the Finishing Period

Composition of grain fed to steers		
Item, % of DM	Control corn	Enogen^®^ corn
Crude protein	8.81	9.11
Starch	71.5	65.3
Starch availability	52.6	53.9
Acid detergent fiber	2.84	4.02
Ether extract	3.20	5.05
Calcium	0.002	0.011
Phosphorous	0.178	0.293
Potassium	0.307	0.468

### RAMS sampling

Collection of RAMS samples followed protocols previously described (Rice et al., 2003) using a sterile foam-tipped swab (VWR, Radnor, PA, USA). Each swab was placed into a sterile tube containing 3 mL of Gram-negative broth (GN Broth; Remel, Lenexa, KS, USA) supplemented with 0.05 mg/L of cefixime (VCC Supplement; Millipore Sigma, St. Louis, MO, USA), 10 mg/L of cefsulodin, and 8 mg/L of vancomycin (GNccv), stored on ice, and transported to the laboratory for further processing. The use of GN broth is recommended for qualitative protocols where selected enrichment is used to detect and/or isolate Gram-negative bacilli from a sample (Remel, 2011).

### Microbiological analyses

RAMS+GNccv sample tubes were processed according to Greenquist et al. (2005) and subjected to immunomagnetic separation (IMS) following previously published methods (Chaney et al., 2018; Sargeant et al., 2003) using an automatic IMS machine (KingFisher™ mL; Thermo Fisher Scientific, Waltham, MA, USA) according to manufacturer's guidelines. A 50-μL aliquot of phosphate buffered saline-Tween 20 containing the resultant bead-bacteria complex was spread-plated on ntChromagar plates (BBL™ CHROMagar™ O157; BD Difco, Franklin Lakes, NJ, USA) supplemented with 5 mg/L of novobiocin (Novobiocin supplement; Thermo Fisher Scientific) and 2.5 mg/L of potassium tellurite (potassium tellurite hydrate; Millipore Sigma) and incubated at 37°C for 18–24 h.

After incubation, 2 colonies suspected as *Escherichia coli* O157:H7 were streaked for isolation on Sorbitol MacConkey agar (CT-SMAC; Remel) supplemented with 0.05 mg/L of cefixime and 2.5 mg/L of potassium tellurite (CT-Supplement; Millipore Sigma) and incubated for 18–24 h at 37°C. A single colony was picked from each plate, transferred into 9 mL tryptic soy broth (BD Difco), incubated for up to 24 h at 37°C, and 1 mL from each tube was frozen in duplicate at −80°C with 10% glycerol. The MicroSEQ™ *Escherichia coli* O157:H7 detection kit (Applied Biosystems, Foster City, CA, USA) was used with an ABI 7500 FAST PCR machine and RapidFinder™ Express Software (Applied Biosystems) to confirm isolates as *Escherichia coli* O157:H7, according to the manufacturer's workflows and parameters.

### Statistical analyses

The PCR result for each animal was a binary response (positive or negative for *Escherichia coli* O157:H7). Because the overall prevalence rate was low (46/955 ≈ 4.8%), data were analyzed using the generalized linear mixed model (GLMM; suitable for data with a large number of positive samples) and exact conditional logistic regression (ECLR; suitable for data with low number of positive samples) (section 7.3 of Agresti, 2013). For the GLMM, fixed effects included sampling date, silage, corn, and silage × corn (interactions involving sampling date were not significant and therefore were removed); the random effect was pen. For the ECLR, fixed effects were corn, silage, and corn × silage; sampling date served as the stratifying variable. All tests were conducted at the 0.05 significance level and, when applicable, were two sided. Statistical analyses were performed using Statistical Analysis Software (SAS version 9.4; Cary, NC, USA) GLIMMIX and LOGISTIC procedures.

## Results

**FIG. 1.**
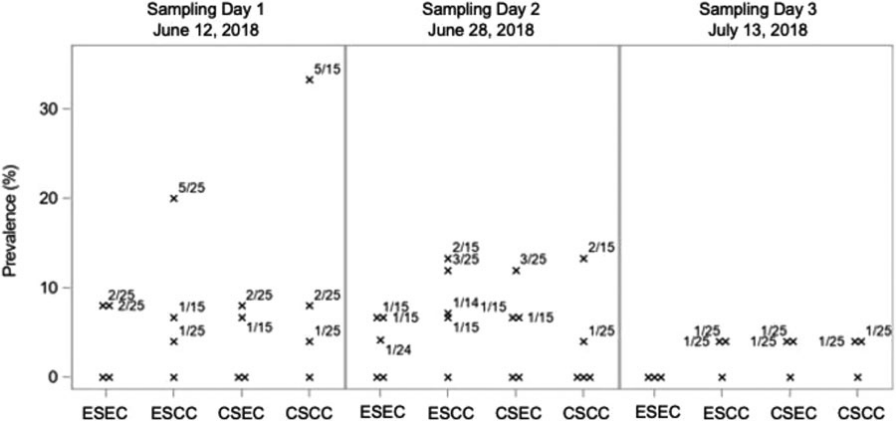
Pen-level summary of *Escherichia coli* O157:H7 binary responses from rectoanal mucosal swab samples collected at re-implant on sampling days 1, 2, and 3 from steers fed each diet. The finishing diet treatments were as follows: CSCC, ESCC, CSEC, and ESEC. Pens with nonzero prevalence are labeled by the corresponding number of positive animals as the numerator and the number of animals in the pen as the denominator. Samples were collected after 62 (sampling day 1; June 12, 2018), 78 (sampling day 2; June 28, 2018), and 93 (sampling day 3; July 13, 2018) days. These sampling dates coincided with re-implanting steers based on harvest days to achieve similar days on final implant before harvest. CSCC, control silage with control corn; CSEC, control silage with Enogen corn; ESCC, Enogen silage with control corn; ESEC, Enogen silage with Enogen corn.

Figure 1 illustrates *Escherichia coli* O157:H7 prevalence on the pen level. The numbers of pens with 0, 1, 2, and 3 positive animals were 20, 18, 6, and 2, respectively. There were two pens with 5 positive animals, suggesting the pen effect might be trivial.

Variance of pen was estimated as 0.22 with a standard error of 0.24. Test of zero variance indicated that there was no significant evidence supporting the presence of pen effect (*p* = 0.13). Hence, fixed effects were estimated, in GLMM, with and without the pen effect. To account for low prevalence, the statistical analysis also applied ECLR that focuses on assessing the difference between diet types, while controlling the effect of sampling date. This approach does not accommodate random effects or estimate prevalence, however. The *p*-values for silage × corn were 0.395 in GLMM with pen effect, 0.394 in GLMM without pen effect, and 0.531 in ECLR. Tables 4 and 5 report results of the analysis for assessing main effects. The nonadjusted *Escherichia coli* O157:H7 prevalence rates are summarized in Table 6.

**Table 4.** Statistical Analysis Summary of *Escherichia coli* O157:H7 Binary Responses from Rectoanal Mucosal Swab Samples Collected from Steers Fed Each Diet Combination of Corn and Silage: The Main Effect of Corn

		Silage-specific prevalence ± SE, %		Across-silage prevalence ± SE, %		Odds ratio to control (p)		
Corn	Silage	With pen effect	Without pen effect	With pen effect	Without pen effect	With pen effect	Without pen effect	ECLR
Enogen^®^	Enogen	2.5 ± 1.0	2.6 ± 1.0	3.0 ± 0.8	3.1 ± 0.8	0.56	0.56	0.57
	Control	3.6 ± 1.3	3.6 ± 1.2			(0.108)	(0.068)	(0.071)
Control	Enogen	5.8 ± 1.7	5.9 ± 1.5	5.2 ± 1.2	5.3 ± 1.1			
	Control	4.7 ± 1.5	4.8 ± 1.4					

Data are analyzed with and without the effect of pen.

ECLR, exact conditional logistic regression; SE, standard error.

**Table 5.** Statistical Analysis Summary of *Escherichia coli* O157:H7 Binary Responses from Rectoanal Mucosal Swab Samples Collected from Steers Fed Each Diet Combination of Corn and Silage: The Main Effect of Silage

		Corn-specific prevalence ±SE, %		Across-corn prevalence ±SE, %		Odds ratio to control (*p*)		
Silage	Corn	With pen effect	Without pen effect	With pen effect	Without pen effect	With pen effect	Without pen effect	ECLR
Enogen^®^	Enogen	2.5 ± 1.0	2.6 ± 1.0	3.8 ± 1.0	3.9 ± 0.9	0.91	0.83	1.00
	Control	5.8 ± 1.7	5.9 ± 1.5			(0.795)	(0.932)	(1.000)
Control	Enogen	3.6 ± 1.3	3.6 ± 1.2	4.4 ± 1.0	4.2 ± 0.9			
	Control	4.7 ± 1.5	4.8 ± 1.4					

Data are analyzed with and without the effect of pen.

ECLR, exact conditional logistic regression; SE, standard error.

**Table 6.** Summary of *Escherichia coli* O157:H7 Binary Responses from Rectoanal Mucosal Swab Samples Collected at Re-Implant on Sampling Days 1, 2, and 3 from Steers Fed Each Diet Combination of Corn and Silage

Silage	Corn	Sampling day 1*^a^* (June 12, 2018)			Sampling day 2*^a^* (June 28, 2018)			Sampling day 3*^a^* (July 13, 2018)			Overall (all sampling dates)		
		N	EC Pos.	Prevalence, %	N	EC Pos.	Prevalence, %	N	EC Pos.	Prevalence, %	N	EC Pos.	Prevalence, %
Enogen^®^	Enogen	80	4	5.0	84	3	3.6	75	0	0.0	239	7	2.9
	Control	80	7	8.8	84	7	8.3	73	2	2.7	237	16	6.8
Control	Enogen	80	3	3.8	85	5	5.9	75	2	2.7	240	10	4.2
	Control	80	8	10.0	84	3	3.6	75	2	2.7	239	13	5.4
Overall		320	22	6.9	337	18	5.3	298	6	2.0	955	46	4.8

Overall study prevalence, including the overall prevalence for each individual sampling day, is also included.

^a^ Samples were collected after 62 (sampling day 1; June 12, 2018), 78 (sampling day 2; June 28, 2018), and 93 (sampling day 3; July 13, 2018) days. These sampling dates coincided with re-implanting steers based on harvest days to achieve similar days on final implant before harvest.

EC Pos., *Escherichia coli* O157:H7 positive samples.

In research studies, the odds ratio of prevalence serves as an efficacy measure that is an alternative to prevalence ratio (Tamhane et al., 2016). The statistical analysis implemented in this study evaluated two levels of each main effect (grain and silage) on the basis of odds ratio. According to Tables 4 and 5, odds of an *Escherichia coli* O157:H7 positive in cattle fed with EC silage was similar to cattle fed CON silage. The odds of *Escherichia coli* O157:H7 in cattle fed EC corn was 0.57, as high as cattle fed with CON corn; in other words, a diet containing EC reduced the odds of *Escherichia coli* O157:H7 prevalence by 43% in comparison to a CON corn diet, although this was not significant (Table 4).

## Discussion

There are several factors that must be considered when evaluating the data described herein, including variability in overall prevalence across the sampling days (Table 6). What is likely the most important consideration is the variability in nutrient content across the experimental diets. Tables 1–3 summarize nutrient composition, including variability between components of control and EC diets. Perhaps the most notable difference is that starch comprised 30.73% and 2.79% of dry matter (DM) in Enogen and control silage, respectively. Table 2 also highlights notable differences between Enogen and control silage for acid detergent fiber and neutral detergent fiber. Differences observed in the composition of silages may be contributed to growing conditions or maturity, which was likely the result of harvesting at the same time to maintain consistency of ensiling duration across CON and EC silages.

It is challenging to conduct silage research due to differences in maturity of corn at the time of harvest. Johnson et al. (2003) observed that as corn maturity increases, starch content increases. Although these differences are significant, corn silage only contributed to 8% of the diet on a DM basis. Rusche et al. (2020) fed EC as corn silage in finishing diets at inclusion rates of 12% or 24% and did not observe an improvement in the performance for silage type; therefore, in our experiment, feeding EC silage at 8% would have little effect on starch digestion. Discussion of diets, and overall impact on *Escherichia coli* O157:H7 shedding in cattle, must be considered in the context of nutrient variability, which may have impacted and/or confounded the results of this study. Specifically, differences in SFC characteristics as it comprised 74.5% (DM basis) of the diet.

Previous data on EC processed as dry-rolled corn (DRC) in finishing diets show an increase in postruminal starch digestion (Jolly-Breithaupt et al., 2016) and improved feed efficiency (Johnson et al., 2019; Johnson et al., 2018); however, data on the influence of EC on total tract starch digestibility, particularly in the case of steam-flaked EC as used in this study, are limited. Steam-flaked EC in this experiment had greater starch availability compared to CON.

Zinn (1990) observed that starch availability increases as bulk density of SFC decreases and tends to linearly increase ruminal digestion, which numerically decreases postruminal starch digestion. In this experiment, corn types were flaked to different bulk densities to achieve similar starch availability, as observed by Horton et al. (2020). Published data demonstrate increased hindgut starch digestion leads to increased hindgut volatile fatty acid (VFA) concentration, making it a less hospitable site for *Escherichia coli* O157:H7 proliferation (Buchko et al., 2000; Fox et al., 2007). Wolin (1969) first reported in 1969 that VFAs are inhibitory to *E. coli*. However, this likely cannot directly explain the reduced odds of *Escherichia coli* O157:H7 observed in cattle fed the EC versus CON corn because EC was fed as SFC in this study, which likely impacts the site of starch digestion.

This is supported by Fox et al. (2007), who reported significantly higher (*p* < 0.001) *Escherichia coli* O157:H7 prevalence in cattle fed SFC (65%), in comparison to DRC (30%). It is unclear if increased alpha amylase activity in steam-flaked EC enhances starch digestion only in the rumen, or if postruminal fermentation is also increased, and likely challenging to discern differences in grain types due to differences in composition of the grain in this experiment. Understanding the impact of steam-flaked EC on *Escherichia coli* O157:H7 in the hindgut of cattle requires further investigation, and should be evaluated as a possible mechanism of action in future studies; however, that was beyond the scope of this study.

This study aimed to understand whether modifying cattle diets using EC grain and/or silage impacts carriage of the foodborne pathogen *Escherichia coli* O157:H7 in cattle, as several studies in the literature report on the impact of diet manipulation on preharvest foodborne pathogen carriage in cattle. Purple prairie clover and sainfoin demonstrated potential anti-*Escherichia coli* O157:H7 action (Liu et al., 2013), with supplements containing seaweed, specifically *Ascophyllum nodosum*, also decreasing the prevalence of *Escherichia coli* O157:H7 (Braden et al., 2004; Wang et al., 2009). More recently, finishing diets containing distiller's grains (DGs), a high protein feed inclusion, resulted in increased shedding of *Escherichia coli* O157:H7 in cattle (Chaney et al., 2018), depending upon DG percentage in the diet (Chaney et al., 2018; Jacob et al., 2008; Wells et al., 2009), demonstrating that diet manipulation can increase pathogen prevalence.

In comparison, this study did not demonstrate an increase in *Escherichia coli* O157:H7 associated with including EC in finishing diets, rather data suggest that EC has a tendency to reduce the odds of *Escherichia coli* O157:H7 prevalence in cattle. Previous observations have reported that EC increases feed efficiency and average daily gain by up to 5.50% and 6.00%, respectively, when fed as grain or silage (Johnson et al., 2019; Johnson et al., 2018; Jolly-Breithaupt et al., 2019). When combined with these study data, this suggests that EC improves animal productivity and does not increase *Escherichia coli* O157:H7 in cattle. However, several limitations have been discussed that limit the interpretation of *Escherichia coli* O157:H7 data presented and these limitations prevent a conclusion regarding impact.

## Conclusions

Data described herein suggest that feeding EC does not increase *Escherichia coli* O157:H7 carriage in cattle, and it may tend to decrease the pathogen. However, nutrient differences between control and EC diets combined with a low prevalence make it challenging to interpret the resultant data. To our knowledge, this was the first study undertaken to determine the impact of feeding EC on *Escherichia coli* O157:H7 prevalence in finishing cattle, and the data are limited in scope. The data described herein provide information (1) for beef and feed grain industries about the potential impacts of feeding EC corn on *Escherichia coli* O157:H7 shedding in cattle, and (2) that can serve as the foundation for future research studies investigating the impact of EC on *Escherichia coli* O157:H7 or other foodborne pathogens in cattle.
